# Salpingectomy versus tubal occlusion in laparoscopic sterilisation (SALSTER): a national register-based randomised non-inferiority trial

**DOI:** 10.1016/j.lanepe.2024.101026

**Published:** 2024-08-11

**Authors:** Annika Strandell, Leonidas Magarakis, Karin Sundfeldt, Mathias Pålsson, Per Liv, Annika Idahl

**Affiliations:** a Department of Obstetrics and Gynecology, Institute of Clinical Sciences, Sahlgrenska Academy, University of Gothenburg, Gothenburg, Sweden; b Region Västra Götaland, Department of Obstetrics and Gynecology, Sahlgrenska University Hospital, Gothenburg, Sweden; c Section of Sustainable Health, Department of Public Health and Clinical Medicine, Umeå University, Umeå, Sweden; d Department of Clinical Sciences, Obstetrics and Gynecology, Umeå University, Umeå, Sweden

**Keywords:** Opportunistic salpingectomy, Tubal occlusion, Tubal ligation, Laparoscopic sterilisation, Epithelial ovarian cancer, Randomised controlled trial

## Abstract

**Background:**

Opportunistic salpingectomy to reduce ovarian cancer incidence has become increasingly common despite the lack of randomised trials investigating its safety. In SALSTER, we tested whether salpingectomy for laparoscopic sterilisation is non-inferior to tubal occlusion regarding complications up to eight weeks postoperatively.

**Methods:**

SALSTER is a register-based randomised non-inferiority trial in which 41 gynaecological departments in Sweden participated. After being reported to The Swedish National Quality Register of Gynaecological Surgery (GynOp) for laparoscopic sterilisation, women aged <50 years received study information and could consent to participation online. If eligible, randomisation was performed by the examining/operating gynaecologist before surgery, with stratification for centre, and allocation 1:1 to salpingectomy or tubal occlusion. Blinding was attempted for patients but was impossible for surgeons. The first primary outcome, any complication up to eight weeks postoperatively, was routinely reported in GynOp through physician assessment of patient questionnaires, medical records and personal contact. Complications up to eight weeks postoperatively, a primary safety outcome, were analysed in the per-protocol population. The non-inferiority margin for the difference in the absolute risk of complications was defined as ten percentage points. Missing data were handled using multiple imputation. SALSTER was registered at ClinicalTrials.gov (NCT03860805).

**Findings:**

Between April 4, 2019, and March 31, 2023, 539 women were randomised to salpingectomy and 527 to tubal occlusion. In the salpingectomy and tubal occlusion arms, 40 and 18 women discontinued their participation in the trial and another 26 and 10 did not receive the allocated surgery, respectively. Calculated on imputed data, any complication up to eight weeks postoperatively occurred in 8.1% (38.5/473) of patients after salpingectomy and in 6.2% (31.0/499) of patients after tubal occlusion. The risk difference was 1.9 percentage points (95% confidence interval −1.4 to 5.3).

**Interpretation:**

Laparoscopic salpingectomy is non-inferior to tubal occlusion regarding complication rates up to eight weeks postoperatively.

**Funding:**

This research was funded by the 10.13039/501100002794Swedish Cancer Society, the Lena Wäppling foundation, the Swedish state under the ALF-agreement, 10.13039/501100004885Umeå University, County of Värmland, and Gothenburg Society of Medicine.

**Research in context**

**Evidence before this study**

Before commencing the SALSTER trial, a systematic literature search was performed on opportunistic salpingectomy at sterilisation for ovarian cancer prevention. The search was updated October 2, 2020, before publication of the review in 2021. All studies comparing salpingectomy for sterilisation and tubal occlusion at laparoscopy or laparotomy were eligible. The terms ‘salpingectomy’, ‘tubectomy’, and ‘Fallopian tube removal’ combined with ‘sterilisation’, ‘tube ligation’, ‘prophylactic’, and their variations were used to search the Cochrane Library, Embase, and PubMed. The only randomised controlled trials (RCTs) were three very small trials on sterilisation during Caesarean section, none at laparoscopy. Three small cohort studies at laparoscopy or laparotomy, with a high or unclear risk of bias, were underpowered for assessing complications. A recent update (February 10, 2024) using the same criteria revealed no additional RCT. One register-based cohort study on opportunistic salpingectomy compared with tubal occlusion at laparoscopic sterilisation reported on proxies of ovarian function. No difference in outcomes related to menopause were noted, but the length of follow-up was too short for this young age group. Two small retrospective cohort studies on laparoscopic sterilisation comparing salpingectomy with tubal occlusion reported on operative time but were too small to assess any adverse events.

**Added value of this study**

SALSTER is the first RCT powered to compare complication rates after laparoscopic tubal occlusion versus opportunistic salpingectomy. Therefore, it fills a knowledge gap by providing high quality evidence concerning the safety of performing opportunistic salpingectomy at laparoscopic sterilisation. Salpingectomy compared with tubal occlusion did not increase the risk beyond a pre-defined margin for complications at surgery and up to eight weeks postoperatively. Women planned for laparoscopic sterilisation and offered salpingectomy, can now be counselled regarding the surgical safety and will be better suited to make informed decisions about removing healthy Fallopian tubes.

**Implications of all the available evidence**

Surgical safety of laparoscopic salpingectomy for sterilisation has now been established and women can be counselled accordingly. A change in practice from tubal occlusion to salpingectomy can be anticipated. However, the issues of salpingectomy affecting ovarian function, early menopause, and its potential negative long-term health consequences still need to be addressed. Knowledge is currently lacking to inform women about these potential risks, but subsequent reporting from SALSTER will provide further insights. Furthermore, the effect size of opportunistic salpingectomy on the reduction of ovarian cancer incidence is not known, and RCTs are not feasible for this rare outcome. Thus, observational register-based studies will contribute to future estimations of the effect on ovarian cancer risk.

## Introduction

High-grade serous ovarian carcinoma (HGSC) is widely thought to originate, in most cases, in the fimbriae of the Fallopian tubes.^1^^,^^2^ Consequently, salpingectomy has arisen as a method of preventing epithelial ovarian cancer (EOC). Salpingectomy is supported by observational studies showing that indicated salpingectomy is associated with a decreased incidence of EOC compared with no surgery.^3^^,^^4^ However, the effect of opportunistic salpingectomy on EOC during hysterectomy or sterilisation compared with no salpingectomy is unknown.

In 2010, British Columbia issued an educational initiative aimed at physicians to consider bilateral salpingectomy during benign hysterectomy and sterilisation.^5^ Similarly, a system-wide practice of opportunistic salpingectomy was initiated in California in 2013.^6^ However, there are safety concerns with opportunistic salpingectomy that have not yet been addressed, such as increased perioperative complications and negatively affected ovarian function, potentially contributing to earlier menopause. For this reason, there was a call in 2016 for randomised trials to assess these issues.^7^

When SALSTER started, there were three small randomised controlled trials (RCTs) comparing salpingectomy with tubal ligation at Caesarean delivery.^8^, ^9^, ^10^ However, none of them could assess complications due to small sample sizes. Three large retrospective cohorts of women sterilised at Caesarean delivery, laparoscopy, open or vaginal surgery found no increase in complications after salpingectomy but reported an increase in bleeding and need for analgesics.^5^^,^^6^^,^^11^ Other cohorts were too small to adequately assess complications.^12^

In 2019, when SALSTER started, sterilisations were performed in Sweden at a rate of 102/100,000 women aged 20–49 years. The predominant technique was laparoscopic tubal coagulation, whereas sterilisation via open abdominal surgery, mainly in connection with Caesarean section, has been at a consistently low level for decades (20–28/100,000).^13^ Three factors enabled a register-based RCT: The Swedish National Quality Register for Gynaecological Surgery (GynOp),^14^ the Swedish network for National Clinical Studies in Obstetrics and Gynaecology (SNAKS),^15^ and the national health registers. GynOp is certified at the highest level according to Swedish Quality Registers,^16^ encompasses all major benign gynaecological surgical procedures and has a complete national coverage for units performing gynaecological surgery. The completeness of surgical interventions is estimated at 88.6% according to linkage with the National Patient Register.^17^ GynOp includes health information, type of surgery, complications, and patient follow-up. Continuous validation is reported on the website.^14^ Registration of complications at hysterectomy and adnexal surgery has recently been validated (unpublished, under revision).^18^

In short, there is a lack of high-quality evidence regarding potential risks of opportunistic salpingectomy, and the preconditions for conducting a register-based RCT in Sweden are favourable. Based on the above, SALSTER aims to study the safety of laparoscopic salpingectomy for sterilisation compared with tubal occlusion. Complications from surgery up to eight postoperative weeks and age at onset of natural menopause will be assessed as primary outcomes in a non-inferiority design. This report presents the first primary outcome, complications up to eight weeks postoperatively, and peri-operative secondary outcomes observed in SALSTER.

## Methods

### Study design

**Fig. 1:**
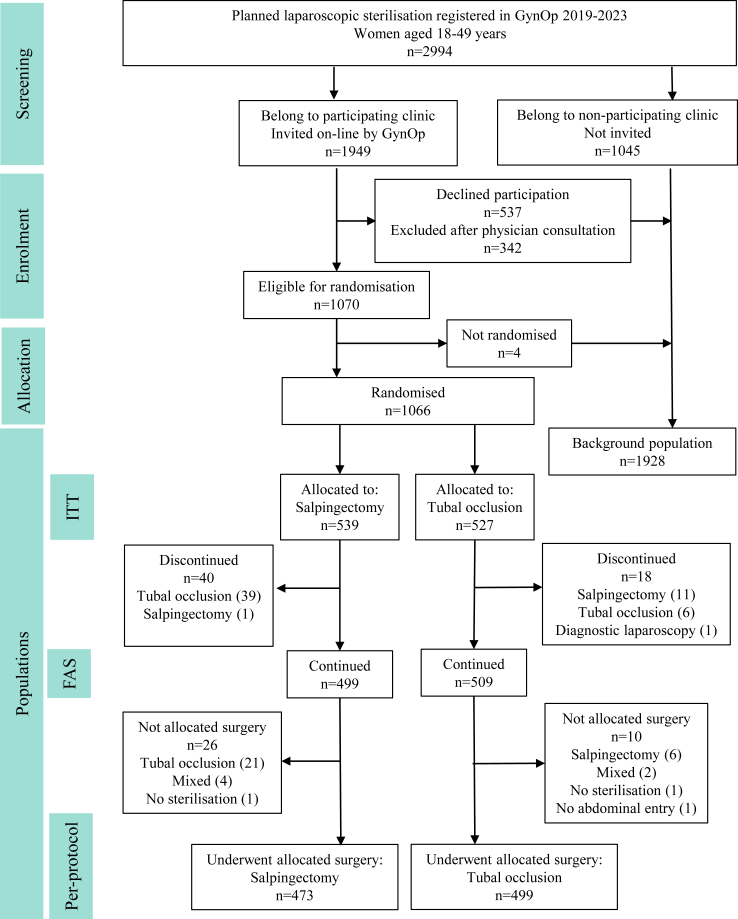
Trial profile.

SALSTER is a nation-wide register-based RCT with a non-inferiority design, using GynOp which encompasses all surgery-performing gynaecological departments in Sweden. Using the SNAKS network and the Swedish Society of Obstetrics and Gynaecology infrastructure to inform about the trial, all 53 centres performing gynaecologic surgery in Sweden were approached and invited to participate. For centres that did not opt out, the SALSTER trial module in GynOp was activated. The trial was approved by the Regional Ethical Review Board in Gothenburg, Sweden, on June 18, 2018 (316-18). The trial was registered at https://clinicaltrials.gov/study/NCT03860805 and the protocol published (Supplementary material).^19^ All women registered in GynOp who were undergoing a sterilisation procedure (Classification of Surgical Procedures LGA or LBE, ICD-10 code Z30.2) during the study period (April 4, 2019, to May 31, 2023) while not participating in SALSTER constituted the background population (Fig. 1), which was used to evaluate the generalisability of the results of the SALSTER trial to the Swedish population.

### Participants

Women under the age of 50 years planned for laparoscopic sterilisation at a participating centre were eligible for inclusion in the study. Exclusion criteria were previous malignancy involving radiation, chemotherapy, or endocrine treatment affecting ovarian function, as well as not understanding the study information available in Swedish. Women with a known hereditary ovarian cancer-related gene mutation would not be eligible for a standard sterilisation procedure and did not fulfil the inclusion criteria.

Oral information about the study was given at the outpatient consultation for the laparoscopic sterilisation procedure. Given that the woman consented to laparoscopic sterilisation, she was registered in GynOp and automatically screened for age eligibility. Potential trial participants received the study information in addition to a standard web-based pre-operative health questionnaire routinely administered by the quality register. Informed consent was mainly given online using multi-factor authentication and stored on the GynOp server, or occasionally by paper in case of technical failure or patient preference.

The routine questionnaire contains items on health status; previous surgeries, including Caesarean section; present medications, including hormonal contraceptives; parity; previous gynaecological disease, including salpingitis; bleeding pattern; endometriosis; pain; dyspareunia; and tobacco use. Weight, length, ASA classification, additional information on diseases, and general and gynaecological status is entered by health care professionals. Study-specific questions concerned age at menarche, additional information on hormonal contraceptives, previous *Chlamydia trachomatis* infections, and the Menopause Rating Scale.^20^ The entire pre-operative questionnaire is provided in the Supplementary material.

### Randomisation and masking

At the final preoperative visit before or on the day of surgery, randomisation to bilateral salpingectomy or no salpingectomy (tubal occlusion) was carried out within a dedicated randomisation module in GynOp. Participants were randomised at a 1:1 ratio using permuted blocks with random sizes of either two or four and stratified for centre. The randomisation performed online by the examining/operating gynaecologist or support staff gave an immediate allocation response. The nature of the trial made blinding of patients difficult and impossible for surgeons. The intention to blind was explained to trial participants, and they were asked to refrain from reading their online medical records until they had responded to the one-year questionnaire. It is uncertain the extent to which this advice was followed; therefore, blinding was unpredictable. Surgeons were not blinded to the intervention when assessing the eight-week questionnaire.

### Procedures and follow-up

Laparoscopic sterilisation was carried out as either salpingectomy or, if allocated to no salpingectomy, any sterilisation method occluding the tube and leaving the fimbriae intact in accordance with local routines and surgeon’s preference, hereafter referred to as tubal occlusion. If unexpected findings were present during surgery, such as adhesions or hydrosalpinx, the decision to deviate from the allocated procedure was at the discretion of the operating surgeon. The procedure that was finally performed was recorded in GynOp in all cases according to established routines. In addition, study-specific variables regarding number, size, and type of trocars, type of instruments and energy used, methods of specimen evacuation, and wound closure techniques were registered. The macroscopic appearance of the pelvic area, uterus, Fallopian tubes, and ovaries was recorded, as well as adhesions, cysts, and tubal pathology. Any intraoperative complications, blood loss, and operative time were also noted.

The surgical procedure performed and complications that occurred up to discharge from the hospital were registered routinely by the physicians directly in GynOp. After discharge, any contact for complications was reported in GynOp. At eight postoperative weeks, the woman received a digital health questionnaire (Supplementary material) asking for any type of complication related to the surgery or symptoms that the woman interpreted as being related to the surgery. If no answer was registered, two automatic digital reminders and subsequent paper reminder were sent to the woman. The average general response rate to the eight-weeks questionnaire during the study period was 87%.^14^ Additional study-specific reminders to participants were included to reduce attrition. In the case of missing data on complications within the study population, the research group contacted the SNAKS representatives to fill out the missing information when possible.

### Outcomes

SALSTER has two primary non-inferiority outcomes. The first reported here is the primary outcome of complications registered in GynOp by the operating surgeon or a consulting gynaecologist and up to eight postoperative weeks. The outcome was based on the physician’s assessment of the existing eight-week patient questionnaire in GynOp, medical records including any postoperative visit and phone contacts if needed. The assessment resulted in a classification of none, mild, or severe complications. Complications were also categorised according to the Clavien-Dindo classification.^21^

The secondary outcomes reported here are complications as assessed by the surgeon according to the Clavien-Dindo classification, severe complications, blood loss, operative time, and length of hospital stay registered at surgery and up to eight weeks postoperatively.

The second primary outcome is age at onset of natural menopause to assess whether tubal occlusion is non-inferior regarding the effect on ovarian function in the long term and will be reported in subsequent publications.

All outcomes of this trial are different aspects of the safety of opportunistic salpingectomy.

### Statistical analysis

SALSTER is designed to declare non-inferiority with at least 80% power for each of its two primary outcomes. Complications up to eight postoperative weeks were calculated to require the largest sample size for reaching sufficient power.^19^ The rate of complications up to eight weeks postoperatively for sterilisation procedures from 2010 to 2017 was 13.6%, specifically retrieved from GynOp in the planning phase of the trial.^14^^,^^19^ An assumption of a true increase in the complication rate of three percentage points (pp) for salpingectomy and a non-inferiority margin of ten pp was based on a clinical expert consensus process within the research group. A calculation based on a one-sided Farrington-Manning test at a 2.5% significance level showed that 411 women were required per randomisation group. To account for a 10% loss to follow-up and a further 5% loss due to randomised women interrupting their participation, the target sample size was determined to be 968.

The non-inferiority hypothesis test was performed as a one-sided test with a significance level of 2.5%. This is equivalent to the upper limit of a two-sided 95% confidence interval (CI) for the difference between salpingectomy and tubal occlusion being lower than the non-inferiority margin.

A superiority hypothesis is appropriate for the long term outcome EOC. However, for complications, we did not expect a lower complication rate after salpingectomy compared with tubal occlusion, but we can accept a somewhat higher complication rate (within the non-inferiority margin) for the benefit of a future decrease in EOC incidence.

The primary analyses were based on the per-protocol population consisting of all women who continued in the trial and were operated according to the allocated procedure (Fig. 1). Secondary analyses were performed on the full analysis set (FAS) according to the intention-to-treat (ITT) principle. The FAS is defined as all randomised women who had surgery and did not discontinue their participation after randomisation. The ITT population is defined as all randomised women who had surgery before May 31, 2023 (i.e., two months after recruitment was closed), also including those who discontinued their participation.

Descriptive statistics for the ITT and FAS populations were calculated by group, presenting mean values and standard deviations, as well as the medians, quartiles, and minimum and maximum values for continuous variables and frequencies and percentages for categorical variables. Descriptive statistics were also calculated for the background population and compared with the FAS population using the Mann–Whitney U test for continuous variables and Pearson’s chi-square test for categorical variables. Furthermore, a withdrawal analysis was conducted comparing the two treatment arms of the FAS with the women who discontinued the trial after randomisation. Statistical tests were conducted using the Kruskal–Wallis test for continuous variables and Pearson’s chi-square test for categorical variables.

A generalised estimation equation (GEE) with a logistic link function and exchangeable covariance structure was used to model the centre-averaged risk of complication by group. The CI for the marginal absolute risk difference between groups was estimated from the GEE model using the delta method and the function *avg_comparisons* from R package *marginaleffects*. As a sensitivity analysis, the unadjusted one-sided 97.5% CI for the difference in complications was calculated using the Farrington-Manning method. The risk ratio (RR) was estimated using a GEE Poisson model with robust standard errors.

The statistical methods in the original study protocol have been updated (December 12, 2022) to adhere to present standards of analysing randomised trials and are included in the statistical analysis plan (SAP) and in the published protocol (Supplementary material).^19^

The original SAP stated that the secondary outcomes were to be investigated using proportional odds models adjusted for centre as a fixed effect. However, due to the many included centres, we deviated from the planned analyses. Operative time was analysed after logarithmic transformation using mixed effects models, adjusting for centre as a random effect. The group difference on the logarithmic scale was retransformed back to natural scale using the exponential function and interpreted as a ratio of the geometric means of operative time. Furthermore, the group difference in arithmetic mean operative time was estimated from the model marginal means using R package *emmeans*. The Clavien-Dindo classifications of complications, blood loss, and length of hospital stay were analysed using ordinal cumulative link functions with centre as a random effect. Blood loss was categorised into classes of 5 ml. Due to many reported null values and a skewed distribution we chose not to estimate absolute differences in blood loss between groups.

Missing variables in the primary analyses were handled using multiple imputation and R package MICE. Using predictive mean matching from 10 donors, 30 imputed data sets were generated and results from analysing each were pooled using Rubin’s rule. Details on the imputation procedure can be found in the Supplementary material. As sensitivity analyses, complete case analyses were performed.

The detailed SAP for the trial is available in the SALSTER registration at ClinicalTrials.gov (NCT03860805). The SAP also contains further details on definitions of the study populations and sample size calculations. For all statistical analyses, R v 4.3.0 was used.

Safety was assessed by an independent Data Safety Monitoring Board as an interim analysis when 50% of the target sample size was reached. Clearance was given for the study to continue recruiting patients.

### Role of the funding source

This work was supported by the Swedish Cancer Society (CAN 21 1408 Pj and CAN 21–848 PJ), the Lena Wäppling foundation, the Swedish state under the agreement between the Swedish government and the county councils, the ALF-agreement (ALFGBG-965130, ALFGBG-965552 and ALF RegVB-969584), Umeå University, Department of Clinical Sciences, and Centre for clinical research, county of Värmland grant number (LIVFOU-929703), The Gothenburg Society of Medicine (GLS-986684).

The funders of the study had no role in the study design, data collection, data analysis, data interpretation, or writing of the report.

## Results

The SALSTER trial randomised women from April 4, 2019, until March 31, 2023. Surgery was performed between April 8, 2019, and May 31, 2023. Out of 53 gynaecological departments in Sweden, 41 representing all levels of care, recruited women for the trial. Three departments actively declined participation. Nine additional departments did not recruit, mainly because they did not offer sterilisations. During the study period, 2994 individuals were eligible for screening (Fig. 1). One-third of women undergoing laparoscopic sterilisation registered in GynOp (n = 1066, 35.6%) were enrolled, leaving 1928 women not enrolled. The Covid-19 pandemic caused an abrupt cease in recruitment, followed by a return to the previous recruitment rate after 18 months (Supplementary Fig. S1). The number of individuals recruited per centre is shown in Supplementary Fig. S2. Following randomisation, 539 women were allocated to salpingectomy and 527 to tubal occlusion, forming the ITT population. Baseline characteristics for the two intervention groups are presented in Table 1.

**Table 1:** Baseline characteristics per randomisation group in the intention-to-treat (ITT) population.

Characteristic	Salpingectomy	Tubal occlusion	Missing S/T
	n = 539	n = 527	
Age (years)	36.2 (5.0)	36.0 (5.4)	0/0
	36 (32; 40)	36 (32, 40)	
	26; 48	25; 49	
BMI (kg/m^2^)	26.7 (5.2)	26.8 (5.2)	41 (8%)/45 (9%)
	25.7 (23.0; 29.4)	26.0 (23.1; 29.7)	
	17.4; 61.3	17.0; 66.4	
Smoking			35 (7%)/45 (9%)
Current	74 (15%)	82 (17%)	
Ex-smoker	195 (39%)	183 (38%)	
Never	235 (47%)	217 (45%)	
Employment	395 (79%)	394 (82%)	36 (7%)/44 (8%)
ASA-classification			5 (1%)/6 (1%)
ASA I	379 (71%)	376 (72%)	
ASA II	149 (28%)	142 (27%)	
ASA III–V	6 (1%)	4 (1%)	
Medical comorbidities			
Hypertension	24 (5%)	19 (4%)	35 (7%)/45 (9%)
Diabetes	6 (1%)	3 (1%)	37 (7%)/47 (9%)
Cardiac condition	4 (1%)	14 (3%)	35 (7%)/44 (8%)
Thrombo-embolism	22 (4%)	26 (5%)	37 (7%)/45 (9%)
Thyroid disease	12 (2%)	6 (1%)	37 (7%)/47 (9%)
Renal disease	11 (2%)	9 (2%)	37 (7%)/47 (9%)
Hepatic disease	8 (2%)	9 (2%)	37 (7%)/47 (9%)
Neurologic disorder	20 (4%)	9 (2%)	49 (9%)/56 (11%)
Mental condition	115 (23%)	95 (20%)	39 (7%)/49 (9%)
Prior abdominopelvic surgery			
Any	165 (32%)	156 (32%)	22 (4%)/36 (7%)
Caesarean section	100 (20%)	91 (19%)	44 (8%)/55 (10%)
Appendectomy	53 (11%)	50 (11%)	46 (8%)/56 (10%)
Adnexal surgery	24 (5%)	23 (5%)	46 (8%)/57 (11%)
Ectopic pregnancy	18 (4%)	21 (4%)	48 (9%)/58 (11%)
Myomectomy	7 (1%)	2 (<1%)	48 (9%)/58 (11%)
Gynaecological history			
Age at menarche	12.5 (1.5)	12.7 (1.6)	158 (29%)/144 (27%)
	12 (12; 13)	13 (12; 14)	
	9; 17	8; 18	
Parity			36 (7%)/44 (8%)
0	58 (12%)	70 (15%)	
1	51 (10%)	50 (10%)	
2	198 (39%)	209 (43%)	
≥3	196 (39%)	154 (32%)	
Prior ectopic pregnancy	25 (5%)	28 (6%)	52 (10%)/65 (12%)
Length of hormonal contraception (months)	12 (7.0)	12 (7.1)	178 (33%)/179 (34%)
	11 (6; 16)	11 (6; 16)	
	0; 30	0; 32	
Lower abdominal pain	104 (21%)	92 (19%)	39 (7%)/47 (9%)
Coital pain (moderate or severe)	26 (6%)	30 (7%)	102 (19%)/88 (17%)
Endometriosis	20 (4%)	13 (3%)	42 (8%)/48 (9%)
Myoma	24 (5%)	11 (2%)	42 (8%)/47 (9%)
Prior salpingitis	7 (1%)	8 (2%)	43 (8%)/48 (9%)
Prior *C. trachomatis* infection	113 (29%)	103 (27%)	149 (28%)/146 (28%)

Continuous variables are expressed as mean (SD), median (Q1; Q3), min; max, and categorical as n (% of valid). Missing values are expressed as n (% of total) in the respective groups; Salpingectomy (S)/Tubal occlusion (T).

Seven percent (n = 40) of women allocated to salpingectomy discontinued their participation, including 39 who underwent tubal occlusion and one who underwent bilateral salpingectomy. Among women allocated to tubal occlusion, 3.5% (n = 18) discontinued participation, including 11 who underwent bilateral salpingectomy and six who underwent tubal occlusion. One patient had a frozen pelvis and did not undergo a sterilisation procedure (Fig. 1). A withdrawal analysis showed similar baseline characteristics among dropouts as among the remaining women who constituted the FAS population (Supplementary Table S1).

Among 499 women in the FAS population allocated to salpingectomy, 26 did not undergo the allocated procedure (Fig. 1). Twenty-one women underwent bilateral tubal occlusion, whereas four underwent unilateral salpingectomy and contralateral tubal occlusion. One woman had a frozen pelvis in which only one tube was accessible, and it was occluded. In the tubal occlusion group of the FAS population, ten out of 509 women did not undergo the allocated procedure. Six women underwent bilateral salpingectomy, whereas two underwent unilateral tubal occlusion and contralateral salpingectomy. One woman had a bowel injury leading to laparotomy without performing a sterilisation procedure, and in one woman the entry procedure was complicated by a massive subcutaneous emphysema and surgery was discontinued before abdominal entry. Thus, the per-protocol population consisted of 473 women who underwent salpingectomy and 499 who underwent tubal occlusion.

The FAS population had a mean age of 36.1 years and a mean BMI of 26.8 kg/m^2^. They were generally healthy, although one-fifth reported a mental condition. One-third of the population had previously undergone abdominopelvic surgery, most commonly Caesarean section. Time from randomisation to surgery had a similar distribution; 60% in both groups were randomised on the day of surgery. The two randomisation groups within the FAS and per-protocol populations had similar baseline characteristics (Supplementary Tables S2 and S3).

The main outcome complication up to eight weeks postoperatively was analysed in the per-protocol population from imputed data. Any complication after salpingectomy was reported in 38.5/473 women (8.1%) and after tubal occlusion in 31.0/499 women (6.2%) (Table 2). The risk difference was 1.9 pp (95% CI -1.4 to 5.3), not exceeding the predefined non-inferiority margin of ten pp. The sensitivity analyses yielded similar risk differences: the risk difference in the unadjusted analysis based on imputed data using Farrrington-Manning's method was 1.9 pp (95% CI -1.4 to 5.4) and, in the complete case analysis with adjustment for centre using GEE, it was 2.1 pp (95% CI -1.3 to 5.4). The results based on complete cases are presented in Supplementary Table S4.

**Table 2:** Outcomes of SALSTER in the per-protocol population based on imputed data.

	Salpingectomy (n = 473)	Tubal occlusion (n = 499)	Difference (95% CI)	p value	Relative treatment effect (95% CI)	p value
**Primary outcome**						
Any complication up to 8 weeks postoperatively	38.5 (8.1%)	31.0 (6.2%)	1.9 percentage points (−1.4, 5.3)	0.26	RR 1.33 (0.82, 2.10)	0.25
**Secondary outcomes**						
Severe complication up to 8 weeks postoperatively	1 (0.2%)	1 (0.2%)	n.c.		n.c.	
Complications classified according to Clavien-Dindo			n.c.		OR 1.35 (0.90, 2.11)	0.078
1	30.2 (6.4%)	23.6 (4.7%)				
2	6.3 (1.3%)	6.3 (1.3%)				
3a	1.0 (0.2%)	0.1 (<0.1%)				
3b	1.0 (0.2%)	1.0 (0.2%)				
4	0	0				
5	0	0				
Perioperative blood loss (ml)	6.7 (11.2) 5 (0; 10)	4.1 (9.4) 0 (0; 5)	n.c.		OR 2.33 (1.81, 3.00)	<0.0001
Operative time (min)	44.6 (18.8) 45 (30; 55)	29.3 (14.9) 26 (19; 37)	15.6 (14.1, 17.1)	<0.0001	GMR 1.56 (1.55, 1.58)	<0.0001
Length of hospitalisation (days)	0.0 (0.1) 0 (0; 0)	0.0 (0.1) 0 (0; 0)	n.c.		OR 0.84 (0.25, 2.78)	0.78

Data are mean (SD), median (Q1; Q3), n (%), n is not an integer due to being a pooled number across the imputed data sets. Differences are reported as salpingectomy - tubal occlusion and relative treatment effects as salpingectomy/ tubal occlusion. RR = risk ratio, OR = odds ratio, GMR = geometric means ratio, n.c. = not calculable.

The distribution of complications according to Clavien-Dindo classification is presented in Table 2. A non-significant higher complication rate in the salpingectomy group was represented mainly by Clavien-Dindo grade 1. Only three women had a grade 3 classification, based on re-operation. The number of severe complications is listed in Table 2. The perioperative blood loss was significantly greater (p < 0.0001) with salpingectomy than with tubal occlusion but minimal in both groups (mean 6.7 versus 4.1 ml) (Table 2). The operative time was longer for salpingectomy than for tubal occlusion (44 versus 29 min, p < 0.0001). The mean reported difference was 16 (95% CI 14 to 18) minutes (Table 2). The length of hospitalisation was measured in days, and most of the cases were discharged within the first postoperative day. The odds ratio for having a longer hospitalisation between salpingectomy and tubal occlusion was 0.84 (95% CI 0.25 to 2.8).

In 60% of all cases, a main trocar >10 mm was used, to accommodate a 10 mm laparoscope. In salpingectomy, two or more accessory trocars were used in 85%, with a >10 mm trocar in up to 30%. When tubal occlusion was performed, one accessory trocar was used in 80%, with a >10 mm trocar in <10%. The most common tubal occlusion technique was coagulation with a bipolar device in the ampullary/isthmic portion of the tube with or without cutting the tubes. Of the salpingectomy procedures, bipolar coagulation was used in 54%, either in combination with cold scissors or other instruments functioning with monopolar current. In 44%, an advanced bipolar sealing device was used for salpingectomy while ultrasonic energy devices were rarely used. Fascia suturing for re-approximation of at least one fascia defect was performed in 71% after salpingectomy and in 60% after tubal occlusion.

Outcomes were also analysed in the FAS population (Supplementary Table S5). The results were similar to those in the primary analysis of the per-protocol population. Comparing the FAS with the background population, no clinically relevant differences were revealed (Supplementary Table S6). The median BMI was 0.6 units higher in the FAS. Ex-smokers were more common in the FAS than in the background population, but current smokers were similarly frequent. Diabetes was uncommon but more often reported in the background population. Complications up to eight weeks postoperatively occurred in 6.8% (101/1488) in the background population.

## Discussion

The main finding of SALSTER is that laparoscopic salpingectomy for sterilisation does not increase the risk of complications beyond a pre-defined margin at surgery and up to eight weeks postoperatively compared with tubal occlusion. As previously reported by our systematic review, no sufficiently large trials comparing the complication risk of laparoscopic salpingectomy versus tubal occlusion for sterilisation have been published.^12^ An update of the systematic literature search was conducted February 10, 2024, and did not reveal any RCT on laparoscopic sterilisation. Among observational studies, Hanley et al. reported on proxies for menopausal symptoms in a register-based study of opportunistic salpingectomy at sterilisation.^22^ No difference in outcomes were noted, though the length of follow-up for menopause was too short for this fairly young age group. Two other retrospective cohort studies on laparoscopic sterilisation comparing salpingectomy with tubal occlusion were too small to conclude on adverse events but reported operative time.^23^^,^^24^ To date, SALSTER is the only RCT designed to assess the safety of laparoscopic salpingectomy for sterilisation.

SALSTER is a pragmatic trial, and the use of GynOp is part of every-day practice; thus, the trial was conducted in line with routine standard care. Laparoscopic salpingectomy in general is a longer and more traumatic procedure than tubal occlusion, as more surgical steps are required. As seen in SALSTER, salpingectomy often requires more abdominal incisions for additional trocars, extra dissection, more extended diathermy, and specimen evacuation compared with tubal occlusion. Thus, some surgeons assume an increase in complications compared with tubal occlusion, whereas others argue that salpingectomy is an easy and standardised procedure. We hypothesised and demonstrated that there is no increased risk of complications during and up to eight weeks after salpingectomy. Low-grade complications according to Clavien-Dindo were non-significantly more common after salpingectomy, potentially reflecting a true difference in grade 1 complications. However, this difference was small and the complications of a mild nature, suggesting limited clinical importance. Severe complications, similar in the two arms, would have been a clinically more interesting outcome but would have required a very large sample size. These complications are often related to laparoscopy per se, the most common being entry complications,^25^ and not related to the planned sterilisation technique.

The use of more resources resulted in a prolonged operative time for salpingectomy, 16 min longer than for tubal occlusion, which is in line with previous reports.^23^^,^^24^ A longer time in the operating theatre and more advanced instrumentation for salpingectomy increases the costs of the sterilisation procedure. This cost could be balanced with potentially reduced costs for EOC treatments. However, high-quality evidence regarding the long-term outcome menopausal age and the effect size of EOC prevention generated by opportunistic salpingectomy are not yet available to inform cost-effectiveness analyses and health economic evaluations.

The SALSTER trial currently provides clinical information about the short-term safety of laparoscopic salpingectomy for sterilisation compared with tubal occlusion. SALSTER will also report on the impact of salpingectomy on menopausal age and measure anti-Müllerian hormone levels in a sub-study. The trial is not powered to analyse the impact on the incidence of EOC. SALSTER will, however, contribute data to a parallel trial, Hysterectomy and OPPortunistic SAlpingectomy (HOPPSA), in which patients undergoing benign hysterectomy will be randomised to salpingectomy or no salpingectomy.^26^^,^^27^ The randomised study populations in HOPPSA and SALSTER, together with the non-randomised population registered in GynOp when undergoing hysterectomy or sterilisation, will be used to estimate the effectiveness of opportunistic salpingectomy on EOC prevention.

The major strengths of SALSTER are the randomised design and sufficiently large sample size to evaluate complications. This register-based RCT design combines the best of two worlds, the randomisation, which counteracts selection bias, and the real-world data from a register. The registration of most variables, including the primary outcome complications, was achieved according to well-established routines, and the inter-rater agreement in GynOp has been reported to be high.^18^ Using a quality register including patient-reported outcomes at a set period followed by physician assessment to capture surgical complications is considered advantageous (unpublished, under revision). Another strength is the nationwide coverage, including all levels of hospitals, improving generalisability, which was demonstrated in the comparison between the randomised cohort and the background population. Despite the decreased availability of sterilisation procedures during the Covid-19 pandemic, recruitment only marginally decreased, and the trial could be completed according to plan. Cooperation with SNAKS representatives at all trial sites facilitated the communication with clinics during recruitment and at data quality assurance before the analysis phase, increasing the quality of the trial and reducing attrition bias.

There are limitations inherent to data from registers. The design of the pre-existing variables was not always optimal. Missing or unreasonable values were seen among important variables. However, we were able to trace and locate the correct information in the medical records with the help of the SNAKS representatives at each trial site. Even though the generalisability is likely to be high, it is probably more so in societies similar to the Swedish setting. Payment regulation for sterilisations may differ between and even within countries. The different health care regions in Sweden expose a variety of charges over time. The financial aspect causing selection bias is counteracted by the randomised design but must be considered when evaluating generalisability. One recognised limitation is the discrepancy between the estimated complication rate from GynOp before the start of the trial (13.6%) and the actual result (8.0%, ITT-population). This difference can be explained by improved surgical skills and techniques over time, as evidenced by a similar complication rate in the background populations during the study period. We must also stress that the primary analysis was based on the per-protocol population that excluded women without allocated procedures but with reported complications. Thus, the trial evaluated the difference between the sterilisation techniques, whereas the baseline complication rate included adverse events related to laparoscopy per se.

Even though blinding was attempted for study participants, it could not be secured because access to medical records is regulated by Swedish law. We assume that the assessment of complications is not likely to be affected by lack of blinding.

The rate of sterilisations at laparoscopy and Caesarean delivery may vary between countries. Salpingectomy performed at the time of Caesarean section is likely to carry additional risks than salpingectomy performed in a non-pregnant woman.^28^ Reasons include pregnancy-induced changes in tissue physiology and increased blood circulation. Thus, the results of this study are unlikely to be applicable to sterilisation procedures at Caesarean section, and separate study designs are needed for the pregnant population.

SALSTER was made possible by the hysteroscopic salpingeal occluding technique (Essure™) being withdrawn from the market due to adverse effects. If the less invasive hysteroscopic approach re-enters the market, a scientific and clinical dilemma will be at hand, as the SALSTER results will not be applicable. Which approach would be offered for sterilisation in the general population if opportunistic salpingectomy, only available at laparoscopy, offers some prevention of EOC? Potential ovarian function impairment caused by salpingectomy also needs to be weighed in the decision but has not been properly addressed.

The results of SALSTER further strengthen the medical evidence towards a change in practice at laparoscopic sterilisation. The short-term safety of salpingectomy has now been confirmed and women can be counselled accordingly. However, the issues of ovarian function, early menopause, and its potential negative long-term health consequences still need to be addressed.^29^, ^30^ SALSTER will provide good quality data on the effect of salpingectomy on age at menopause, but the results will not be available for another 10–15 years. Concerning the long-term effects of opportunistic salpingectomy on ovarian cancer incidence, prospective observational studies that can sufficiently control for confounding will probably provide the evidence needed.

## Contributors

AS is the principal investigator of SALSTER and conceived the study. AS and AI planned the design and wrote the initial study protocol. PL is responsible for the statistical analyses and wrote the SAP together with AS and AI. AI and MP have worked in close connection with the GynOp register and, together with AS and LM, developed the SALSTER-specific parts of the questionnaires. Register data were handled by PL, LM, and AS, and the final analyses were conducted by PL. All authors contributed different parts to the first draft of the manuscript and KS, AI, AS, and PL revised it for intellectual content. All authors had full access to all data in the study and had final responsibility for the decision to submit for publication. All authors have read and approved the final version of the manuscript.

## Data sharing statement

Data used in the present study was extracted from the Swedish National Quality Register of Gynaecological Surgery (GynOp) after an application to the GynOp steering group, accompanied by approval from the Swedish Ethical Review Authority. The data cannot be shared publicly because the individual-level data contain potentially identifying and sensitive patient information and cannot be published due to legislative and ethical review restrictions (https://etikprovningsmyndigheten.se).

## Declaration of interests

We declare no competing interests.

## Appendix A Supplementary data

Supplementary Figs. S1 and S2 and Tables S1–S6

Published SALSTER protocol

Questionnaires
