# Supplementary material for: Salpingectomy versus tubal occlusion in laparoscopic sterilisation (SALSTER): a national register-based randomised non-inferiority trial
Source: Lancet Reg Health Eur. 2024 Aug 11;45:101026. doi: 10.1016/j.lanepe.2024.101026 (PMC11838100; doi:10.1016/j.lanepe.2024.101026)
Supplement: Supplementary Figs. S1 and S2 and Tables S1–S6 [file mmc1.pdf]

Content Supplementary material

	Page
Figure S1: Accumulated number of women randomised per quartile year	1
Figure S2: Total number of women randomised per centre	1
Table S1: Withdrawal analysis comparing baseline characteristics in women who discontinue and continue after randomisation	2
Table S2: Baseline characteristics per randomisation group in the full analysis set (FAS) population	3
Table S3: Baseline characteristics per randomisation group in the per-protocol population	4
Table S4: Outcomes of SALSTER in the per-protocol population based on complete case data	5
Table S5: Outcomes of SALSTER in the full analysis set (FAS) population based on imputed data	6
Table S6: Comparison of baseline characteristics of the full analysis set (FAS) of the SALSTER trial and the background population having laparoscopic sterilisation in Sweden	7
Description of imputation procedure	8

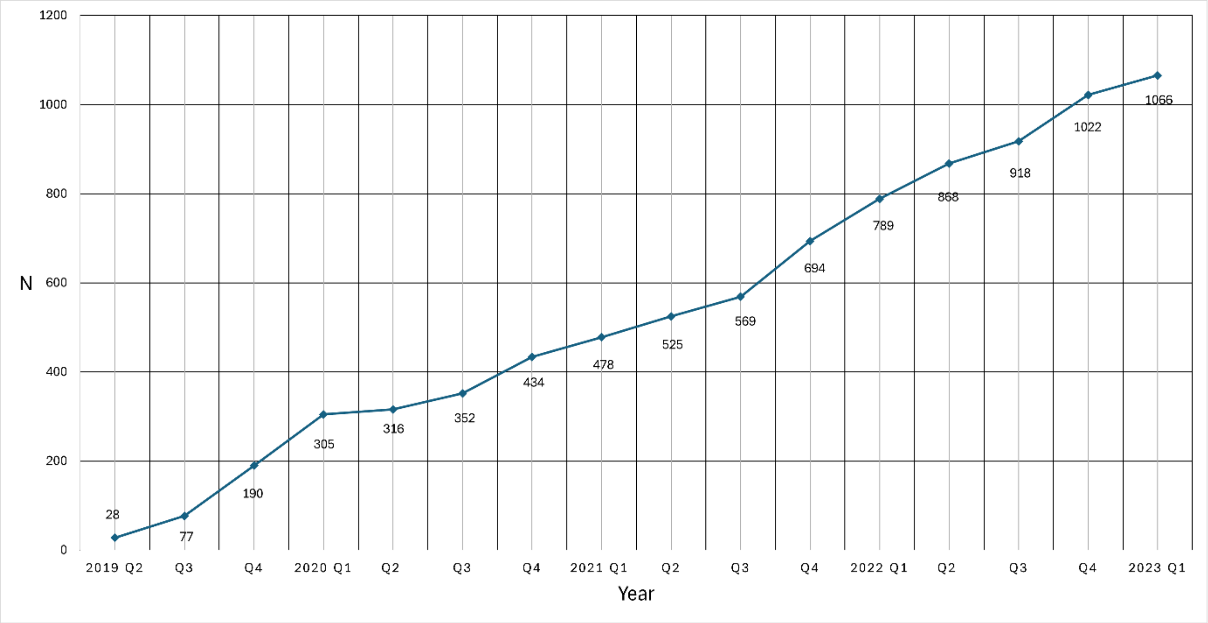

Figure S1: **Accumulated number of women randomised per quartile year**

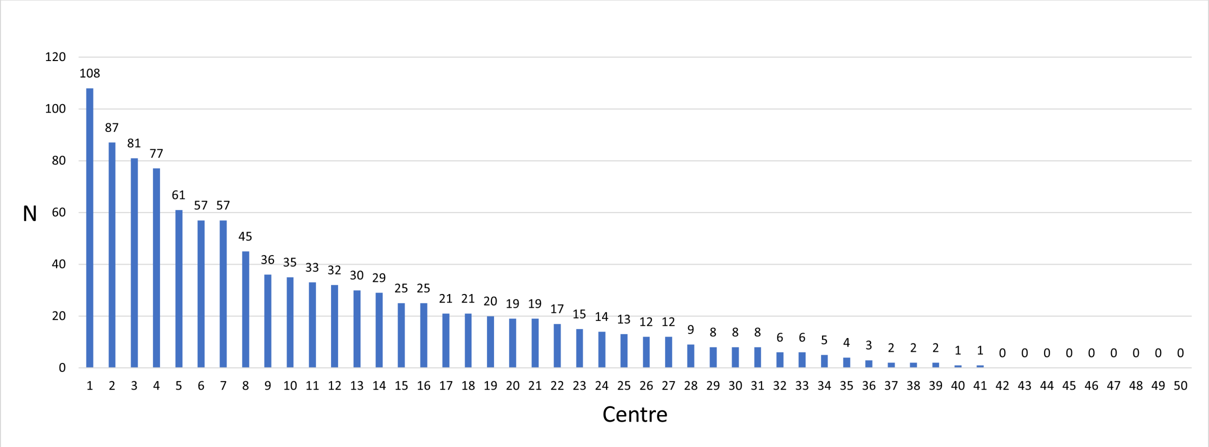

Figure S2: **Total number of women randomised per centre**

	Allocated to Salpingectomy		Allocated to Tubal occlusion		
**Characteristic**	**Discontinued n=40**	**Continued n=499**	**Discontinued n=18**	**Continued n=509**	**P value**
Age (years)	36·0 (32·0; 38·3)	36·0 (32·0; 41·0)	34·5 (31·0; 39.8)	36.0 (32.0; 40.0)	0.68
BMI (kg/m^2)	24.5 (23·4; 27·7)	25·9 (23·0; 29·7)	25·6 (24·0; 27·6)	26·1 (23·1; 29·7)	0·50
missing	3	38	3	42	
Smoking					0·50
Current	8 (22%)	66 (14%)	2 (13%)	80 (17%)	
Former	9 (24%)	186 (40%)	6 (40%)	177 (38%)	
Never	20 (54%)	215 (46%)	7 (47%)	210 (45%)	
missing	3	32	3	42	
Employment	28 (76%)	367 (79%)	12 (80%)	382 (82%)	0·61
missing	3	33	3	41	
Prior abdominopelvic surgery*	17 (47%)	148 (32%)	5 (33%)	151 (33%)	0·33
missing	4	38	3	49	
Hypertension	1 (3%)	23 (5%)	1 (7%)	18 (4%)	0.67
missing	3	32	3	4	
Diabetes	0	6 (1%)	0	3 (1 %)	§
missing	3	34	3	44	
Cardiac condition	0	4 (1%)	0	14 (3%)	§
missing	3	32	3	42	
Mental condition	8 (22%)	107 (23%)	4 (27%)	91 (20%)	0.55
missing	3	36	3	46	
Parity					†
0	3 (8%)	55 (12%)	3 (20%)	67 (14%)	
1	7 (19%)	44 (9%)	2 (13%)	48 (10%)	
2	16 (43%)	182 (39%)	7 (47%)	202 (43%)	
≥3	11 (30%)	185 (40%)	3 (20%)	151 (32%)	
missing	3	33	3	41	
Prior ectopic pregnancy	2 (6%)	23 (5%)	3 (20%)	25 (6%)	0·13
missing	6	46	3	62	
Endometriosis	2 (5%)	18 (4%)	0	13 (3%)	0·56
missing	3	39	3	45	
Myoma	1 (3%)	23 (5%)	1 (7%)	10 (2%)	0·064
missing	3	39	3	44	
Previous salpingitis	1 (3%)	6 (1%)	0	8 (2%)	0·64
missing	3	40	3	45	

The p-value is calculated from a test of the null hypothesis of all four groups being equal. Continuous variables are expressed as median (Q1; Q3) and dichotomous as n (% of valid). Missing values are expressed as n. *Including Caesarean section, appendectomy, adnexal surgery, myomectomy and ectopic pregnancy. †Test was found not meaningful due to many cells in cross table. §Test was found not meaningful due to few cases in cells.

***TABLE S1*: Withdrawal analysis comparing baseline characteristics in women who discontinue and continue after randomisation**

Characteristic	Salpingectomy	Tubal occlusion	Missing S/T
	n=499	n=509	
Age (years)	36·3 (5·1)	36·0 (5.4)	0/0
	36 (32; 41)	36 (32; 40)	
	26; 48	25; 49	
BMI (kg/m^2)	26·8 (5·3)	26·8 (5·2)	38 (8%)/42 (8%)
	25·9 (23·0; 29·7)	26·1 (23·1; 29·7)	
	17·4; 61·3	17·0; 66·4	
Smoking			32 (6%)/42 (8%)
current	66 (14%)	80 (17%)	
ex-smoker	186 (40%)	177 (38%)	
never	215 (46%)	210 (45%)	
Employment	367 (79%)	382 (82%)	33 (7%)/41 (8%)
ASA-classification			5 (1%)/5 (1%)
ASA I	354 (72%)	363 (72%)	
ASA II	135 (27%)	137 (27%)	
ASA III-V	5 (1%)	4 (1%)	
Medical comorbidities			
Hypertension	23 (5%)	18 (4%)	32 (6%)/42 (8%)
Diabetes	6 (1%)	3 (1%)	34 (7%)/44 (9%)
Cardiac condition	4 (1%)	14 (3%)	32 (6%)/42 (8%)
Thrombo-embolism	20 (4%)	25 (5%)	34 (7%)/42 (8%)
Thyroid disease	11 (2%)	6 (1%)	34 (7%)/44 (9%)
Renal disease	9 (2%)	8 (2%)	34 (7%)/43 (8%)
Hepatic disease	8 (2%)	8 (2%)	34 (7%)/44 (9%)
Neurologic disorder	18 (4%)	9 (2%)	44 (9%)/53 (10%)
Mental condition	107 (23%)	91 (20%)	36 (7%)/46 (9%)
Prior abdominopelvic surgery			
Any	148 (32%)	151 (33%)	38 (8%)/49 (10%)
Caesarean section	89 (19%)	87 (19%)	40 (8%)/53 (10%)
Appendectomy	48 (11%)	49 (11%)	42 (8%)/53 (10%)
Adnexal surgery	23 (5%)	23 (5%)	42 (8%)/54 (11%)
Ectopic pregnancy	16 (4%)	19 (4%)	44 (9%)/55 (11%)
Myomectomy	6 (1%)	2 (<1%)	44 (9%)/55 (11%)
Gynaecological history			
Age at menarche	12·5 (1·5)	12·7 (1·6)	140 (28%)/134 (26%)
	12 (11; 13)	13 (12; 14)	
	9; 17	8; 18	
Parity			33 (7)/41 (8%)
0	55 (12%)	67 (14%)	
1	44 (9%)	48 (10%)	
2	182 (39%)	202 (43%)	
≥3	185 (40%)	151 (32%)	
Prior ectopic pregnancy	23 (5%)	25 (6%)	46 (9%)/62 (12%)
Length of hormonal contraception (months)	12·0 (7·0)	12·0 (7·0)	159 (32%)/167 (33%)
	11 (6; 17)	11 (6; 16)	
	0; 30	0; 32	
Lower abdominal pain	91 (20%)	84 (18%)	36 (7%)/45 (9%)
Coital pain (moderate or severe)	26 (6%)	29 (7%)	88 (18%)/85 (17%)
Endometriosis	18 (4%)	13 (3%)	39 (8%)/45 (9%)
Myoma	23 (5%)	10 (2%)	39 (8%)/44 (9%)
Prior salpingitis	6 (1%)	8 (2%)	40 (8%)/45 (9%)
Prior *C. trachomatis* infection	103 (28%)	102 (27%)	132 (24%)/137 (24%)

Continuous variables are expressed as mean (SD), median (Q1; Q3), min; max, and dichotomous as n (% of valid). Missing values are expressed as n (% of total) in the respective groups; Salpingectomy (S)/Tubal occlusion (T).

Table S2: **Baseline characteristics per randomisation group in the full analysis set (FAS) population**

Characteristic	Salpingectomy	Tubal occlusion	Missing S/T
	n=473	n=499	
Age (years)	36·2 (5·0)	35·9 (5.4)	0/0
	36 (32; 40)	36 (32; 40)	
	26; 48	25; 49	
BMI (kg/m^2)	26·6 (5·0)	26·8 (5·2)	34(7%)/42 (9%)
	25·7 (22·9; 29·3)	26·1 (22·9; 29·3)	
	17·3; 46·9	17·0; 66·4	
Smoking			28 (6%)/42 (8%)
current	59 (13%)	79 (17%)	
ex-smoker	180 (41%)	174 (38%)	
never	206 (46%)	204 (45%)	
Employment	350 (79%)	373 (81%)	29 (6%)/41 (8%)
ASA-classification			5 (1%)/5 (1%)
ASA I	336 (72%)	354 (72%)	
ASA II	127 (27%)	136 (28%)	
ASA III-V	5 (1%)	4 (1%)	
Medical comorbidities			
Hypertension	20 (4%)	18 (4%)	28 (6%)/42 (8%)
Diabetes	6 (1%)	3 (<1%)	29 (6%)/44 (9%)
Cardiac condition	3 (1%)	14 (3%)	28 (6%)/42 (8%)
Thrombo-embolism	18 (4%)	23 (5%)	30 (6%)/42 (8%)
Thyroid disease	11 (2%)	6 (1%)	29 (6%)/44 (9%)
Renal disease	9 (2%)	7 (2%)	34 (7%)/43 (9%)
Hepatic disease	8 (2%)	8 (2%)	29 (6%)/44 (9%)
Neurologic disorder	17 (4%)	9 (2%)	38 (8%)/53 (11%)
Mental condition	101 (23%)	91 (20%)	32 (7%)/46 (9%)
Prior abdominopelvic surgery			
Any	140 (31%)	149 (32%)	17 (4%)/33 (7%)
Caesarean section	84 (19%)	86 (19%)	37 (8%)/52 (10%)
Appendectomy	44 (10%)	47 (11%)	39 (8%)/53 (11%)
Adnexal surgery	22 (5%)	23 (5%)	39 (8%)/53 (11%)
Ectopic pregnancy	15 (3%)	19 (4%)	41 (7%)/54 (11%)
Myomectomy	6 (1%)	2 (<1%)	41 (9%)/54 (11%)
Gynaecological history			
Age at menarche	12·4 (1·5)	12·7 (1·6)	132 (28%)/131 (26%)
	12 (11; 13)	13 (12; 14)	
	9; 17	8; 18	
Parity			30 (6%)/41 (8%)
0	49 (11%)	64 (14%)	
1	43 (10%)	47 (10%)	
2	172 (39%)	198 (43%)	
≥3	179 (40%)	149 (33%)	
Prior ectopic pregnancy	21 (5%)	25 (5%)	43 (9%)/62 (12%)
Length of hormonal contraception (months)	12·1 (7)	12 (7)	148 (31%)/164 (33%)
	11 (6; 17)	11 (6; 16)	
	0; 30	0; 32	
Lower abdominal pain	85 (19%)	83 (18%)	33 (7%)/45 (9%)
Coital pain (moderate or severe)	24 (6%)	28 (7%)	80 (17%)/82 (16%)
Endometriosis	16 (4%)	13 (3%)	35 (7%)/45 (9%)
Myoma	23 (5%)	10 (2%)	35 (7%)/44 (9%)
Prior salpingitis	6 (1%)	7 (2%)	36 (8%)/45 (9%)
Prior *C. trachomatis* infection	99 (28%)	101 (27%)	119 (25%)/120 (24%)

Continuous variables are expressed as mean (SD), median (Q1; Q3), min; max, and dichotomous as n (% of valid). Missing values are expressed as n (% of total) in the respective groups; Salpingectomy (S)/Tubal occlusion (T).

Table S3: **Baseline characteristics per randomisation group in the per-protocol population**

	Salpingectomy (n=473)	Tubal occlusion (n=499)	Difference (95% CI)	p value	Relative treatment effect (95% CI)	p value
**Primary outcome**						
Any complication up to 8 weeks postoperatively	37 (8·2%)	29 (6·1%)	2·1 percentage points (-1·3, 5·4)	0·23	RR 1·33 (0·83, 2·14)	0·23
**Secondary outcomes**						
Severe complication up to 8 weeks postoperatively	1 (0·2%)	1 (0·2%)	n.c.		n.c.	
Complications classified according to Clavien-Dindo			n.c.		OR 1·43 (0·83, 2·14)	0·18
1	29 (6·4%)	21 (4·4%)				
2	6 (1·5%)	6 (1·3%)				
3a	1 (0·2%)	0 (0.0%)				
3b	1 (0·2%)	1 (0·2%)				
4	0	0				
5	0	0				
Perioperative blood loss (ml)	6·7 (11·2)<br>5 (0; 10)	4·1 (9·4)<br>0 (0; 5)	n.c.		OR 2·33 (1·81, 2·99)	<0·0001
Operative time (min)	44.6 (18·8)<br>45 (30; 55)	29·3 (14·9)<br>26 (19; 37)	15·5 (13·3, 17·7)	<0.0001	GMR 1·56 (1·49, 1·64)	<0·0001
Length of hospitalisation (days)	0 (0·1)<br>0 (0; 0)	0 (0·1)<br>0 (0; 0)	n.c.		OR 0·84 (0·25, 2·81)	0·774

Data are mean (SD), median (Q1; Q3), n (%). Differences are reported as salpingectomy - tubal occlusion and relative treatment effects as salpingectomy / tubal occlusion. RR=risk ratio, OR=odds ratio, GMR=geometric means ratio, n.c.=not calculable. For information on number of missing data per putcome, see Supplementary appendix on imputations.

Table S4: **Outcomes of SALSTER in the per-protocol population based on complete case data**

	Salpingectomy (n=499)	Tubal occlusion (n=509)	Difference (95% CI)	p value	Relative treatment effect (95% CI)	p value
**Primary outcome**						
Any complication up to 8 weeks postoperatively	42·1 (8·4%)	33·0 (6·5%)	2·0 percentage points (-1·4, 5·3)	0·25	RR 1·30 (0·83, 2·05)	0·25
**Secondary outcomes**						
Severe complication up to 8 weeks postoperatively	1 (0·2%)	2 (0·4%)	n.c.		n.c.	
Complications classified according to Clavien-Dindo			n.c.		OR 1·33 (0·83, 2·14)	0·23
1	32·4 (6·5%)	24·2 (4·8%)				
2	7·6 (1·5%)	6·5 (1·3%)				
3a	1·1 (0·2%)	0·0 (0.0%)				
3b	1·0 (0·2%)	2·2 (0·4%)				
4	0	0				
5	0	0				
Perioperative blood loss (ml)	6·6 (11·2)<br>0 (0; 10)	4·1 (9·3)<br>0 (0; 5)	n.c.		OR 2·3 (1·76, 2·87)	<0·0001
Operative time (min)	44·5 (18·8)<br>30 (30; 55)	29·7 (15·9)<br>19 (19; 37)	15·0 (13·6, 16·5)	<0.0001	GMR 1·54 (1·52, 1·55)	<0·0001
Length of hospitalisation (days)	0·0 (0·1)<br>0 (0; 0)	0·0 (0·2)<br>0 (0; 0)	n.c.		OR 0·84 (0·26, 2·78)	0·78

Data are mean (SD), median (Q1; Q3), n (%), n is not an integer due to being a pooled number across the imputed data sets. Differences are reported as salpingectomy - tubal occlusion and relative treatment effects as salpingectomy / tubal occlusion. RR=risk ratio, OR=odds ratio, GMR=geometric means ratio, n.c.=not calculable.

Table S5: **Outcomes of SALSTER in the full analysis set population (FAS) based on imputed data**

Characteristic	FAS	Background	P value	Missing F/B
	**n=1008**	**n=1928**		
Age (years)	36·1 (5·2)	36·3 (5·1)	0·25	0/0
	36·0 (32·0; 40·0)	36·0 (32·0; 40·0)		
BMI (kg/m^2)	26·8 (5·2)	26·3 (4·9)	0·013	80 (8%)/328 (12%)
	26·0 (23·1; 29·7)	25·4 (22·6; 29·1)		
Smoking			0·018	74 (7%)/318 (16%)
current	146 (16%)	263 (16%)		
ex-smoker	363 (39%)	537 (33%)		
never	425 (46%)	810 (50%)		
Employment	749 (80%)	1287 (80%)	0·88	74 (7%)/318 (16%)
ASA-classification			0·71	10 (1%)/15 (1%)
ASA I	717 (72%)	1383 (72%)		
ASA II	272 (27%)	507 (27%)		
ASA III-V	9 (1%)	23 (1%)		
Medical comorbidities				
Hypertension	41 (4%)	64 (4%)	0·63	74 (7%)/328(17%)
Diabetes	9 (1%)	34 (2%)	0·030	78 (8%)/327 (17%)
Cardiac condition	18 (2%)	26 (2%)	0·56	74 (7%)/315 (16%)
Thrombo-embolism	45 (5%)	60 (4%)	0·18	76 (8%)/315 (16%)
Hepatic disease	16 (2%)	28 (2%)	0·96	78 (8%)/328 (17%)
Mental condition	198 (21%)	311 (19%)	0·24	82 (8%)/329 (17%)
Prior abdominopelvic surgery*	299 (32%)	502 (32%)	0·69	87 (9%)/344 (18%)
Gynaecological history				
Parity			0·51	74 (7%)/319 (17%)
0	122 (13%)	223 (14%)		
1	92 (10%)	151 (9%)		
2	384 (41%)	619 (38%)		
≥3	336 (36%)	616 (38%)		
Prior ectopic pregnancy	48 (5%)	77 (5%)	0·76	74 (7%)/404 (21%)
Lower abdominal pain	175 (19%)	267 (17%)	0·13	81 (8%)/313 (16%)
Coital pain (moderate or severe)	55 (7%)	72 (5%)	0·13	173 (17%)/503 (26%)
Endometriosis	31 (3%)	49 (3%)	0·71	84 (8%)/339 (18%)
Myoma	33 (4%)	70 (4%)	0·32	83 (8%)/333 (17%)
Prior salpingitis	14 (2%)	35 (2%)	0·23	85 (8%)/336 (17%)

Continuous variables are expressed as mean (SD), median (Q1; Q3), and dichotomous as n (% of valid). Missing values are expressed as n (% of total) in the respective groups; FAS (F)/Background (B). *Including Caesarean section, appendectomy, adnexal surgery, myomectomy and ectopic pregnancy.

Table S6: **Comparison of baseline characteristics of the full analysis set (FAS) of the SALSTER trial and the background population having laparoscopic sterilisation in Sweden**

DESCRIPTION OF IMPUTATION PROCEDURE

Multiple imputations by chained equations (MICE) were performed using the mice function from R package mice[1]. The imputations were conditioned on the following variables:

**Predictor variables**	**Scale**	**Imputed values by group S/T**
Group	Dichotomous (salpingectomy or tubal occlusion)	0
Any complication up to 8 weeks postoperatively		25 (5%)/27 (5%)
Age (years)	Continuous	0
BMI (kg/m^2)	Continuous	39 (7%)/45 (9%)
Perioperative blood loss (ml)	Continuous	39 (7%)/45 (9%)
Operative time (min)	Continuous	6 (1%)/10 (2%)
Prior salpingitis	Dichotomous (yes or no)	43 (8%)/48 (9%)
Adhesion	Dichotomous (yes or no)	0 /3 (1%)
Prior abdominopelvic surgery	Dichotomous (yes or no)	38 (8%)/49 (10%)
ASA classification	Three categories (I, II, III-V)	5 (1%)/6 (1%)
Smoking	Four categories (Never, former, 1-5cig/day, >5 cig/day)	36 (7%)/44 (8%)
Imputed variables not used as predictors:		
Complications classified according to Clavien-Dindo	Data available in four categories (1, 2, 3a, 3b)	25 (5%)/28 (5%)
Severe complications up to 8 weeks postoperatively	Dichotomous (yes or no)	25 (5%)/27 (5%)
Perioperative blood loss	Continuous	0 /1 (<1%)

S= Salpingectomy, T=Tubal occlusion

Complications classified according to Clavien-Dindo and *severe complications* were omitted as predictors due to their strong collinearity with *any complications*, and *perioperative blood loss* due to its heavily skewed distribution, making modelling challenging.

Imputations were performed using the *mice* function from R package *mice.*[1] Predictive mean matching from 10 potential donors was used for generating 30 imputed data sets. The function *char2seed()* from R package *TeachingDemos*[2] was used for generating the seed for the pseudo-randomness in the procedure, with the string "SALSTER" as argument.

References

1. Stef van Buuren, Karin Groothuis-Oudshoorn (2011). mice: Multivariate Imputation by Chained Equations in R. Journal of Statistical Software, 45(3), 1-67. DOI 10.18637/jss.v045.i03.
2. Snow G (2020). _TeachingDemos: Demonstrations for Teaching and Learning_. R package version 2.12, <https://CRAN.R-project.org/package=TeachingDemos>
