# Supplementary material for: Salpingectomy versus tubal occlusion in laparoscopic sterilisation (SALSTER): a national register-based randomised non-inferiority trial
Source: Lancet Reg Health Eur. 2024 Aug 11;45:101026. doi: 10.1016/j.lanepe.2024.101026 (PMC11838100; doi:10.1016/j.lanepe.2024.101026)
Supplement: Questionnaires [file mmc3.pdf]

Content Supplementary material (questionnaires)

Pre-operative questionnaire (17 pages)	
Eight-week post-operative questionnaire (10 pages)	

Dear

Kind regards and thanks in advance

DO NOT COPY

Questionnaire prior to surgery

Personal identity number:
Name
Address
Postal code City
Phone
Email

⇦ ***If the above information is missing or incorrect, please fill in here***

Your questionnaire can also be found online. It is easier for us if you complete the questionnaire online.
Go to www.gynop.se, click "LOG IN".
Your password is

There are gaps in the numbering of questions since certain questions are not relevant for you.

DO NOT COPY

1. Date when the questionnaire is completed: ................-.........-........

2. Do you know what surgical procedure is planned for you? ☐ Yes ☐ No

If yes: what procedure ....

....

3a. **Rank** the following reasons for why you have sought medical care. **Enter** 1 for the **most important** reason that you have sought care, 2 for second most important reason, etc.

Write the numbers in the boxes. E.g. [1] [3] [2]

☐ Pain
☐ Bleeding
☐ Pressure and heaviness (e.g. feeling of heaviness, pressure on the bladder, pressure on the intestines)
☐ Prolapse (protrusion from the vagina)
☐ Urinary leakage/urinary incontinence
☐ Childlessness
☐ Other symptoms/reasons? Specify ....

....

3b. How long have you had the problem you ranked as number 1, the most important reason for seeking medical care? Number of years ...., months .... or days............

4. Do you have pain in the pelvic area/lower abdomen?

☐ No

☐ Yes Specify the severity of your pain

- Menstrual cramps, regular monthly cramps
  The pain lasts between .... and .... days.
  - ☐ No, no pain
  - ☐ Yes, mild pain
  - ☐ Yes, moderate pain
  - ☐ Yes, severe pain
  - ☐ Yes, unbearable pain
- Abdominal pain
  - ☐ No, no pain
  - ☐ Yes, mild pain
  - ☐ Yes, moderate pain
  - ☐ Yes, severe pain
  - ☐ Yes, unbearable pain
- Pelvic pain, describe....................................
  ....
  ....
  - ☐ No, no pain
  - ☐ Yes, mild pain
  - ☐ Yes, moderate pain
  - ☐ Yes, severe pain
  - ☐ Yes, unbearable pain

5a. Have you had menstrual periods/vaginal bleeding during the past year? ☐ No ☐ Yes

5b Are your menstrual periods regular? ☐ Yes ☐ No

5c Do you experience spotting/unexpected vaginal bleeding? ☐ Yes ☐ No

5d Do you take hormones that regulate your period? ☐ Yes ☐ No

5e. How would you describe this bleeding?

☐ No menstruation/cessation of menstruation
☐ Light
☐ Moderate
☐ Heavy
☐ Very heavy
☐ Varies from time to time

5f. What treatment have you had over the past 3 years for bleeding?

☐ No treatment
☐ Treated with iron supplements because of abnormal blood test (anemia)
☐ Treatment to regulate monthly periods (oral contraceptives, progesterone – taken approximately 10-12 days a month)
☐ Treatment to reduce the quantity of bleeding during menstruation (medications taken only during menstruation)
☐ Treatment to eliminate menstruation if possible (injections, hormonal IUD, daily tablets)
☐ Other, specify ....

DO NOT COPY

5g. Have you used or are you using a hormonal IUD

☐ No
☐ Don't know
☐ Yes, use it now
☐ Yes, but it was removed

6. How long ago did you have your last menstrual period/vaginal bleeding?

☐ Less than 6 weeks ☐ 6 weeks – 6 months ☐ 7 months – 1 year ☐ More than 1 year

7. Do you have or have you had menopausal symptoms (flushing, sweating, palpitations)?

☐ No
☐ Don't know
☐ Yes

8. Do you take hormones containing oestrogen?

☐ No
☐ Yes, for menopausal symptoms
☐ Yes, for pelvic problems
☐ Yes, for problems with urine/urinary tract
☐ Yes, for another reason ....

9a. Do you have a feeling that something is bulging out from the vagina?

☐ Never ☐ Almost never ☐ 1–3 times per month ☐ 1–3 times per week ☐ Daily

9b. Do you experience chafing in the genital area?

☐ Never ☐ Almost never ☐ 1–3 times per month ☐ 1–3 times per week ☐ Daily

9c Do you use a pessary to prevent prolapse?

☐ No, have never had a pessary
☐ No, not now, but have previously had a pessary
☐ Yes, I have a pessary now
☐ Don't know

DO NOT COPY

10a Do you have problems emptying your bladder?

☐ Never ☐ Almost never ☐ 1–3 times per month ☐ 1–3 times per week ☐ Daily

10b Have you had problems with urinary urgency (sudden onset of a strong need to urinate)?

☐ Never ☐ Almost never ☐ 1–3 times per month ☐ 1–3 times per week ☐ Daily

10c Do you need to get up at night to urinate?

☐ Never ☐ Almost never ☐ Usually one time ☐ Usually two times ☐ More than two times

10d Do you experience urinary leakage or involuntary urination?

☐ Never ☐ Almost never ☐ 1–3 times per month ☐ 1–3 times per week ☐ Daily

If you answered Never – Almost never to question 10d above, go to question 12

Mark an X next to **one of the options** for each question 11a – 11j

11a Do you experience leakage of urine when you get up out of bed? ☐ Yes ☐ No

11b How often do you experience leakage of urine associated with physical activity, or when you laugh, cough or sneeze?

☐ Never
☐ 1–4 times per month
☐ 1–6 times per week
☐ Once a day
☐ More than once a day

11c. How much urine usually leaks with physical activity, or when you laugh, cough or sneeze? *(The question is asked so that we can estimate the quantity of urine leaked).*

☐ No leakage
☐ Damp underwear
☐ Wet underwear
☐ Soaks through clothing
☐ Runs down legs or onto floor

DO NOT COPY

11d. How often do you experience a sudden onset of a strong need to urinate, and leak urine before you reach the toilet?

☐ Never
☐ 1–4 times per month
☐ 1–6 times per week
☐ Once a day
☐ More than once a day

11e. How much urine usually leaks when you have urinary urgency (sudden onset of a strong need to urinate)?*(The question is asked so that we can estimate the quantity of urine leaked).*

☐ No leakage
☐ Damp underwear
☐ Wet underwear
☐ Soaks through clothing
☐ Runs down legs or onto floor

11f. Does urine leak both with physical activity (e.g. cough, heavy lifting, exercise) and with urinary urgency (sudden onset of a strong need to urinate)? ☐ Yes ☐ No

If yes, which one is most problematic?
☐ Leakage with physical activity causes greater discomfort than leakage with urgency
☐ Leakage with urgency causes greater discomfort than leakage with physical activity
☐ Equal discomfort from leakage with urgency and leakage with physical activity

11g. Do you avoid activities (e.g. physical exercise or going out) because you are afraid of leakage?

☐ Never ☐ Seldom ☐ Occasionally ☐ Frequently ☐ Always

11h. Do you avoid places and situations where you know it is difficult to find a toilet?

☐ Never ☐ Seldom ☐ Occasionally ☐ Frequently ☐ Always

11i. Does your urinary leakage affect

your holidays?	☐ Yes	☐ No
your family life?	☐ Yes	☐ No
your sex life?	☐ Yes	☐ No
your social life (going out, meeting friends, etc.)?	☐ Yes	☐ No
your sleep at night?	☐ Yes	☐ No
your working life?	☐ Yes	☐ No

DO NOT COPY

12a Do you ever have problems emptying your bowels?

☐ Never ☐ Almost never ☐ 1–3 times per month ☐ 1–3 times per week ☐ Daily

12b Do you ever have to push against the back wall of the vagina to empty your bowels?

☐ Never ☐ Almost never ☐ 1–3 times per month ☐ 1–3 times per week ☐ Daily

13a. Do you have problems holding in stool or gas?

- ☐ No
- ☐ Yes

If you answered no to the above question, skip to question <u>14a</u>

13b. Do you ever pass gas even when it is inappropriate?

- ☐ Never
- ☐ Almost never
- ☐ Yes, 1-3 times a month
- ☐ Yes, 1-3 times a week
- ☐ Yes, daily

13c. Do you experience leakage of loose stool?

- ☐ Never
- ☐ Almost never
- ☐ Yes, 1-3 times a month
- ☐ Yes, 1-3 times a week
- ☐ Yes, daily

13d. Do you experience leakage of firm stool?

- ☐ Never
- ☐ Almost never
- ☐ Yes, 1-3 times a month
- ☐ Yes, 1-3 times a week
- ☐ Yes, daily

DO NOT COPY

13e. Do you use sanitary pads/protection because of stool leakage?

- ☐ Never
- ☐ Almost never
- ☐ Yes, 1-3 times a month
- ☐ Yes, 1-3 times a week
- ☐ Yes, daily

13f. Does your leakage problem affect your lifestyle?

- ☐ Never
- ☐ Almost never
- ☐ Yes, 1-3 times a month
- ☐ Yes, 1-3 times a week
- ☐ Yes, daily

15a. Have you had intercourse during the past 3 months?

☐ Yes ☐ No ☐ Not applicable ☐ Prefer not to answer

15b. If yes on question 15a, do you experience pain in the genital area during intercourse?

- ☐ No, no pain
- ☐ Yes, mild pain
- ☐ Yes, moderate pain
- ☐ Yes, severe pain
- ☐ Yes, unbearable pain

15c If yes on question 15a, do you experience leakage of urine during intercourse? ☐ Yes ☐ No

15d. If yes on question 15a, do you feel

that your vaginal opening is too small/narrow?	☐ Yes	☐ No
that your vaginal opening is too large/open?	☐ Yes	☐ No
pain in the vaginal opening?	☐ Yes	☐ No
other symptoms from the vaginal opening?	☐ Yes	☐ No

If yes, what type of symptoms? ……………………………………………………………

……………………………………………………………………………………………

DO NOT COPY

IN ORDER TO ASSESS YOUR SITUATION AND OPTIMALLY PLAN YOUR GYNECOLOGICAL TREATMENT WE NEED SOME GYNECOLOGICAL BACKGROUND INFORMATION

16a How many times have you been pregnant? ….....……b. Number of deliveries ….....……

c. Of which number of C-sections ….....……. d. Number of miscarriages ….....……

e.. Number of ectopic pregnancies ….....……

17. Are you pregnant now? ☐ No ☐ Yes ☐ Don't know

18. Has a doctor informed you that you have or you have had any of these diseases/problems?

☐ No ☐ Yes

If yes, specify which one(s) Mark no if you have not had any

Pelvic Inflammatory Disease?	☐ No ☐ Yes
Endometriosis ("chocolate cysts")?	☐ No ☐ Yes
Ovarian cysts?	☐ No ☐ Yes
Abnormal cervical cells?	☐ No ☐ Yes
Myoma/fibroids?	☐ No ☐ Yes
Other?	☐ No ☐ Yes

19a. Have you had any of the following operations? ☐ No ☐ Yes

If yes, specify which one(s) Mark no if you have not had any

Uterine scraping (dilation and curettage, D and C) for haemorrhage, miscarriage or abortion?	☐ No ☐ Yes
Cervical abnormality?	☐ No ☐ Yes
Caesarean section?	☐ No ☐ Yes
Sterilisation?	☐ No ☐ Yes
Ectopic pregnancy?	☐ No ☐ Yes
Cysts, abnormalities of the ovaries/fallopian tube?	☐ No ☐ Yes
Myoma, fibroids?	☐ No ☐ Yes
Hysterectomy (surgical removal of uterus)?	☐ No ☐ Yes
Urinary incontinence?	☐ No ☐ Yes
Prolapse?	☐ No ☐ Yes
Other genital/gynaecological surgical procedure?	☐ No ☐ Yes
Appendectomy?	☐ No ☐ Yes
Other abdominal surgery?	☐ No ☐ Yes

DO NOT COPY

19b. Have you had any **other surgeries** (not pelvic/abdominal)?

☐ No

☐ Yes. Specify what surgery ....

....

FOR ANAESTHESIA/NUMBING AND OTHER CARE PLANNING WE ALSO NEED ANSWERS TO QUESTIONS THAT ARE NOT RELATED TO GYNAECOLOGY

20. Are you gainfully employed? ☐ No ☐ Yes, I work as …………………………………………….

My job is:
☐ Physically demanding
☐ Not physically demanding

21. Are you on sick leave?

☐ Yes, because of the reason for my surgery
☐ Yes, though I am on sick leave for reasons unrelated to my scheduled surgery
☐ No, I am not on sick leave

22a. How tall are you? .... cm b. How much do you weigh? .... kg

23. Do you smoke?

☐ Yes, 1-5 cigarettes daily
☐ Yes, 6-20 cigarettes daily
☐ Yes more than 20 cigarettes daily
☐ No, have never smoked
☐ No, quit in ...(year)

24. Do you usually experience motion sickness and/or seasickness? ☐ No ☐ Yes

25a. Have you ever had a **serious** allergic reaction to any medicinal product (medicine) that resulted in an emergency visit to the doctor?

☐ No ☐ Yes ☐ Don't know

If yes, describe what you reacted to and how you reacted:
....
....

DO NOT COPY

25b. Do you have any mild allergies to medicines?

☐ No ☐ Yes

If yes, describe what you react to and how you react:
....

....

25c. Have you ever had a **serious** allergic reaction to any food, pollen, perfume, etc. that resulted in an emergency visit to the doctor?

☐ No ☐ Yes

If yes, describe what you reacted to and how you reacted:
....

....

25d. Do you have any mild allergies to any food, pollen, perfume, etc?

☐ No ☐ Yes

If yes, describe what you react to and how you react:

....

....

26. Do you or does anyone in your family have any hereditary illness (e.g.. porphyria, hereditary muscle diseases or malignant hyperthermia)?

☐ No ☐ Yes ☐ Don't know

27. Do you have any of the following problems?

☐ No ☐ Yes

If yes, please specify

Easily get nosebleeds	☐ No ☐ Yes
Bleed longer than 10 minutes from small wounds	☐ No ☐ Yes
Large bruises	☐ No ☐ Yes

28. Has a doctor diagnosed you with a blood clot? ☐ No ☐ Yes

If yes, where was the clot located?

....

DO NOT COPY

29. a. Do you have to stop and rest when walking up two flights of stairs? ☐ No ☐ Yes
b. Do you have to stop and rest when walking up a half flight of stairs? ☐ No ☐ Yes

30. Has a doctor now or ever diagnosed you with heart disease? ☐ No ☐ Yes

If yes, please specify

Heart failure	☐ No ☐ Yes
Myocardial infarction (heart attack)	☐ No ☐ Yes
Angina pectoris	☐ No ☐ Yes
Myocarditis (inflammation of the heart muscle)	☐ No ☐ Yes
Valvular heart disease	☐ No ☐ Yes
Cardiac arrhythmia/dysrhythmia, atrial fibrillation	☐ No ☐ Yes
Other heart disease	☐ No ☐ Yes

31a. Has a doctor diagnosed you with lung disease? ☐ No ☐ Yes

If yes, please specify
COPD (chronic obstructive pulmonary disease) ☐ No ☐ Yes
Asthma? ☐ No ☐ Yes
Other pulmonary disease? ☐ No ☐ Yes

31b.Do you have symptoms from the airways or the lungs? ☐ No ☐ Yes

If yes, which of the following:
Persistent cough over the past six months? ☐ No ☐ Yes
Whistling or hissing sound sometimes when I breathe? ☐ No ☐ Yes
Other airway symptoms? ☐ No ☐ Yes

32. Do you have problems from the stomach or intestines? ☐ No ☐ Yes

If yes, please specify:
Diarrhea? ☐ No ☐ Yes
Vomiting/heartburn? ☐ No ☐ Yes
Severe pain? ☐ No ☐ Yes
Constipation? ☐ No ☐ Yes
Other problems? ☐ No ☐ Yes

DO NOT COPY

33a. Has a doctor diagnosed you with any of the following diseases? ☐ No ☐ Yes

If yes, please specify	Enter "No" for those you have not had
Cerebral haemorrhage?	☐ No ☐ Yes
High blood pressure?	☐ No ☐ Yes
Stroke?	☐ No ☐ Yes
Kidney problems?	☐ No ☐ Yes
Goitre?	☐ No ☐ Yes
Diabetes?	☐ No ☐ Yes
Liver/biliary disease?	☐ No ☐ Yes
Jaundice?	☐ No ☐ Yes

33b. Has a doctor diagnosed you with any of the following diseases? ☐ No ☐ Yes

If yes, please specify	Enter "No" for those you have not had
Blood disease?	☐ No ☐ Yes
Joint disease?	☐ No ☐ Yes
Rheumatism?	☐ No ☐ Yes
Muscle disease?	☐ No ☐ Yes
Neurological diseases? (e.g. epilepsy, MS)?	☐ No ☐ Yes
Mental problems?	☐ No ☐ Yes
Other?	☐ No ☐ Yes
....	

34a. Have you been hospitalised at any time during the past six months? ☐ No ☐ Yes

If yes, enter the number of times you have been hospitalised: ....

If yes, at what hospital(s) and for what problem?

....

34b. Over the past 6 months, have you:

sought care from a doctor or dentist abroad? ☐ No ☐ Yes
been treated for multiresistant bacteria? ☐ No ☐ Yes

DO NOT COPY

35. Do you take any medicine regularly (including painkillers, spray, eye drops, insulin injections, oral contraceptives, herbal/natural remedies)?

☐ No ☐ Yes

If you answered Yes, enter the name, strength and how often you take this medicine below.

Name of medicine	Strength of medicine	How often do you take it?

36. Have you taken cortisone tablets over the past three months? ☐ No ☐ Yes

37. Have you had general or other anaesthesia previously? ☐ No ☐ Yes
If yes, were there any problems? ☐ No ☐ Yes

If yes, describe: ....

....

38. Do you have any infectious disease that is transmitted through contact with blood (e.g. HIV or hepatitis)?

☐ No ☐ Yes

39 Are you being treated, or have you have been treated, for cancer? ☐ No ☐ Yes

40 Do you have any of the following?

Urinary catheter or other tube in your body? ☐ No ☐ Yes
Leg ulcers? ☐ No ☐ Yes
Eczema? ☐ No ☐ Yes

41 Do you have problems opening your mouth wide, e.g. at the dentist? ☐ No ☐ Yes

42. Has any family member had problems with general or other anaesthesia?

☐ No ☐ Yes ☐ Don't know

DO NOT COPY

43. If you entered any diseases or surgeries in answer to any of the questions, would you consent to allow us to read the relevant medical records before your surgery?

☐ No ☐ Yes

44. It is also important to know whether you have any other needs that could affect your care. Do you have any of the following problems/needs? ☐ No ☐ Yes

**If yes**, I have	
aches and/or pain	☐ No ☐ Yes
impaired hearing	☐ No ☐ Yes
impaired vision	☐ No ☐ Yes
physical disability	☐ No ☐ Yes
need for interpreter	☐ No ☐ Yes

45. Have you had any problems understanding any question(s) in the questionnaire? ☐ No ☐ Yes
If Yes, write the number of the question and describe the problem:

....

....

....

....

46. Is there anything else that you consider to be important that we should know?

....

....

....

....
Name (of the person who filled in the questionnaire)

DO NOT COPY

SALPINGECTOMY AT STERILIZATION

SALSTER

Below are additional questions relevant to the SALSTER trial.

1. At what age did you have your first period? _________ years of age.

2. If you have given birth, how long have you been breastfeeding in total? _________ months.

Enter the number of months ('0' if you have not breastfed).

3. Have you ever used hormonal contraception (birth control pills, hormonal intrauterine device (IUD), contraceptive patch, contraceptive ring, contraceptive injections, contraceptive implant)?

- Yes. Go to question 3b

- No. Go to question 4

3b. How long in total have you used hormonal contraceptives (pill, IUD, patch, ring, injection, implant)?

Enter the number of years. _________ years.

4. Have you ever had a Chlamydia infection?
- Yes

- No

- Do not know

Menopause Rating Scale (MRS)

Which of the following symptoms apply to you at this time? Please, mark the appropriate box for each symptom. For symptoms that do not apply, please mark 'none'.

**Symptoms:**	**none**	**mild**	**moderate**	**severe**	**very severe**
**Score =**	**0**	**1**	**2**	**3**	**4**
1. Hot flushes, sweating (episodes of sweating)	☐	☐	☐	☐	☐
2. Heart discomfort (unusual awareness of heart beat, heart skipping, heart racing, tightness)	☐	☐	☐	☐	☐
3. Sleep problems (difficulty in falling asleep, difficulty in sleeping through, waking up early)	☐	☐	☐	☐	☐
4. Depressive mood (feeling down, sad, on the verge of tears, lack of drive, mood swings)	☐	☐	☐	☐	☐
5. Irritability (feeling nervous, inner tension, feeling aggressive)	☐	☐	☐	☐	☐
6. Anxiety (inner restlessness, feeling panicky)	☐	☐	☐	☐	☐
7. Physical and mental exhaustion (general decrease in performance, impaired memory, decrease in concentration, forgetfulness)	☐	☐	☐	☐	☐
8. Sexual problems (change in sexual desire, in sexual activity and satisfaction)	☐	☐	☐	☐	☐
9. Bladder problems (difficulty in urinating, increased need to urinate, bladder incontinence)	☐	☐	☐	☐	☐
10. Dryness of vagina (sensation of dryness or burning in the vagina, difficulty with sexual intercourse)	☐	☐	☐	☐	☐
11. Joint and muscular discomfort (pain in the joints, rheumatoid complaints)	☐	☐	☐	☐	☐

Dear

You had surgery/received treatment at our clinic 2 months ago.

DO NOT COPY

Your opinion of the operation
(about 8 weeks)

Personal identity number:
Name
Address
Postal code City
Phone
Email

⇦ ***If the above information is missing or incorrect, please fill in here***

**Your questionnaire can also be found online.
It is easier for us if you complete the questionnaire online.
Go to www.gynop.se, click "LOG IN".
Your password is**

There are gaps in the numbering of questions since certain questions are not relevant for you.

1. Date when the questionnaire is completed: ................-.........-........

2. How do you feel about the length of your hospital stay?

   ☐ Just right
   ☐ Too long
   ☐ Too short

3. Did you need to take pain relieving medicine because of your surgery, after you left the hospital?

   ☐ No
   ☐ Yes How many days?

4. Have you had any vaginal bleeding resulting from your surgery?

   ☐ No
   ☐ Yes. How many days? ………………..

   How heavy was the bleeding?
   ☐ None/insignificant
   ☐ Light
   ☐ Moderate
   ☐ Heavy
   ☐ Very heavy

DO NOT COPY

5a. Do you have problems emptying your bladder?

☐ Never ☐ Almost never ☐ 1–3 times per month ☐ 1–3 times per week ☐ Daily

5b. Have you had problems with urinary urgency (sudden onset of a strong need to urinate)?

☐ Never ☐ Almost never ☐ 1–3 times per month ☐ 1–3 times per week ☐ Daily

5c. Do you need to get up at night to urinate?

☐ Never ☐ Almost never ☐ Usually one time ☐ Usually two times ☐ More than two times

5d. Do you experience urinary leakage or involuntary urination?

☐ Never ☐ Almost never ☐ Usually one time ☐ Usually two times ☐ More than two times

6a. Have you been on sick leave **because of** your surgery?

☐ No, I have not been on sick leave
☐ I was on sick leave for another reason at the time of my surgery, for which reason I cannot answer the question
☐ Yes, I am still on sick leave
☐ Yes, I was on sick leave

6b. **If** you were on sick leave, what date did you return to work in the same capacity as before your surgery?

.... - .... - ....
year month day

6c. **If** you were on sick leave, how do you fell about the total length of the sick leave?

☐ Too long
☐ Just right
☐ Too short
☐ I was on sick leave for another reason at the time of my surgery, for which reason I cannot answer the question

7. How many days after surgery did you need before you could carry out normal activities of daily living and manage **on your own** without more help than before the operation (e.g. personal hygiene, cooking food for yourself, making the bed, taking short walks)? ....days.

8. How do you feel about the surgical outcome so far? My condition is:

☐ Greatly improved
☐ Improved
☐ Unchanged
☐ Worse
☐ Much worse

DO NOT COPY

Following surgery, some problems are normal, transient and expected because you have had an operation. Some people experience prolonged problems after surgery.

9. During the time since you returned home after surgery until the present, have you experienced new problems/complications related to the operation?

☐ No, **skip to question 17**
☐ Yes, mild
☐ Yes, severe/serious
☐ Yes, mild and severe/serious

11a. Did you need to seek medical care during the period after surgery because of these problems/complications?

☐ No ☐ Yes

**If yes**, what medical facility did you visit?	Name of department and hospital/primary care centre that you visited
☐ The department where you had surgery<br><br>☐ Primary care centre<br><br>☐ Other medical facility	....<br><br>..

11b. If yes, when was the first time you visited the medical facility after you returned home?

☐ Within 1 week ☐ 1 to 2 weeks ☐ 2 to 4 weeks ☐ Later than 4 weeks

Treatment of complications and problems

11c. If you answered yes to question 11a: Were you hospitalised because of the complication?

☐ No, I left the hospital the same day
☐ Yes, stayed for one night.
☐ Yes, stayed for two or more nights

11d. If you answered yes to question 11a: Did treatment of the complication include surgery?

☐ No ☐ Yes

DO NOT COPY

11e. If you answered yes to question 11a: Were your problems/complications treated?
Select one or more responses

☐ No
☐ Yes, monitored with additional recheck and/or investigation
☐ Yes, pain relieving medicine
☐ Yes, the wound dressing was changed
☐ Yes, other treatment. Describe: ....

....

11f. If you answered yes to question 11a: Did the complication cause your sick leave to be extended?

☐ No
☐ Yes, number of weeks ………………..

Describe your problems/complications by choosing one or more of the following options.

12a. Tick the affected organ/parts of the body:

☐ Surgical wound
☐ Blood vessels
☐ Uterus
☐ Nerve/Sensation
☐ Urinary bladder
☐ Ureter from the kidney to the urinary bladder
☐ Urethra
☐ Vagina
☐ Intestines
☐ Other (Describe in question 13f)

12b. Did the complication result in any of the following problems:

☐ Rupture of the surgical wound to the abdominal cavity that required a new operation (wound dehiscence)
☐ Abnormal connection to the vagina, intestine or urinary bladder (fistula)
☐ None of the above problems

DO NOT COPY

Different types of complications.

13a. Bleeding:

- ☐ Heavy/prolonged vaginal bleeding
- ☐ Bleeding from abdominal wall/abdominal wound
- ☐ Bleeding from the abdominal cavity
- ☐ Anaemia
- ☐ Other bleeding (Describe in question 13f)
- ☐ No bleeding

13b. Infection:

- ☐ Fever more than 38° for more than 2 days
- ☐ Urinary tract infection
- ☐ Vaginal infection, foul-smelling discharge
- ☐ Infection of surgical wound
- ☐ Uterine infection
- ☐ Infection inside the abdominal cavity (abdominal abscess)
- ☐ General blood poisoning (sepsis)
- ☐ Other infection (Describe in question 13f)
- ☐ Hospitalised because of infection
- ☐ No infection

If you ticked any option for infection, was the infection treated with an antibiotic/penicillin?
☐ No
☐ Yes

13c. Pain:

- ☐ Pain in the abdomen, stomach
- ☐ Genital pain
- ☐ Pubic bone pain
- ☐ Groin pain
- ☐ Pain located elsewhere (Describe in question 13f)
- ☐ No pain

DO NOT COPY

13d. Urination problems:

Select one or more responses

☐ Difficulties emptying the bladder that required treatment

☐ Residual urine test (measurement of urine remaining in the bladder after urination) on several occasions (approximately how many) ....

☐ Urinary catheterisation on several occasions (approximately how many) ....

☐ Self-catheterization for several days (approximately how many) ....

☐ Catheterisation for several days (approximately how many) ....

☐ Other treatment for several days….., specify what treatment ....

☐ Pain on urination persisting more than 1 month after surgery

☐ Problems holding in urine (urinary incontinence)

☐ Other (Describe in question 13f)

☐ No urination problems

13e. General medical complication:

☐ Abnormal fatigue, lethargy

☐ Bowel obstruction (ileus/subileus)

☐ Severe constipation

☐ Blood clot (thrombosis, emboli) with regular monitoring of blood thinners

☐ Other complication (Describe in question 13f)

☐ None of the above

13f. Describe the problems/complications you ticked above**:** ....

....

....

....

....

DO NOT COPY

14. Do you still have problems with anything that you specified in questions 9-13?

☐ No
☐ Yes

If yes, describe: ....

....

15. If you answered yes to any of questions 9-14, would you consent to allow us to read the relevant medical records before your surgery?

☐ No
☐ Yes

16a. Do you have problems holding in stool or gas?

☐ No

☐ Yes

If you answered No to the above question, skip to question <u>17a</u>

16b. Do you ever pass gas even when it is inappropriate?

☐ Never

☐ Almost never

☐ Yes, 1-3 times a month

☐ Yes, 1-3 times a week

☐ Yes, daily

16c. Do you experience leakage of loose stool?

☐ Never

☐ Almost never

☐ Yes, 1-3 times a month

☐ Yes, 1-3 times a week

☐ Yes, daily

16d. Do you experience leakage of firm stool?

☐ Never

☐ Almost never

☐ Yes, 1-3 times a month

☐ Yes, 1-3 times a week

☐ Yes, daily

16e. Do you use sanitary pads/protection because of stool leakage?

☐ Never

☐ Almost never

☐ Yes, 1-3 times a month

☐ Yes, 1-3 times a week

☐ Yes, daily

DO NOT COPY

16f. Does your leakage problem affect your lifestyle?

☐ Never

☐ Almost never

☐ Yes, 1-3 times a month

☐ Yes, 1-3 times a week

☐ Yes, daily

17. Do you have any additional problems related to the surgical procedure:

☐ No
☐ Yes

Describe the problem(s): ....

....

....

....

18a. Have you been called in or will you be called in for a repeat visit/recheck resulting from your surgery?
☐ Yes
☐ No
☐ Don’t know

18b. If No or Don't know, do you need someone from the Department of Obstetrics and Gynaecology to contact you?

☐ No, I will get in touch if any problems arise.
☐ Yes, I would like someone to contact me regarding …………………………………………

DO NOT COPY

19. Have you had any problems understanding any question(s) in the questionnaire? ☐ No ☐ Yes
If Yes, write the number of the question and describe the problem:

....

....

....

....

....

Name (of the person who filled in the questionnaire)

DO NOT COPY
